# Exercise for Older Adults Improves the Quality of Life in Parkinson’s Disease and Potentially Enhances the Immune Response to COVID-19

**DOI:** 10.3390/brainsci10090612

**Published:** 2020-09-06

**Authors:** Mary-Frances E. Hall, Frank C. Church

**Affiliations:** Department of Pathology and Laboratory Medicine, The University of North Carolina at Chapel Hill, School of Medicine, Chapel Hill, NC 27599, USA; mary-france_hall@med.unc.edu

**Keywords:** Parkinson’s disease, COVID-19, SARS-CoV-2, older adults, exercise, pro-immune response, anti-inflammatory response, antiviral, neurodegenerative disorder

## Abstract

Parkinson’s disease (PD) is a progressive neurodegenerative disorder brought about due to dopaminergic neuronal cell loss in the midbrain substantia nigra pars compacta region. PD presents most commonly in older adults and is a disorder of both motor and nonmotor dysfunction. The novel SARS-CoV-2 virus is responsible for the recent COVID-19 pandemic, and older individuals, those with preexisting medical conditions, or both have an increased risk of developing COVID-19 with more severe outcomes. People-with-Parkinson’s (PwP) of advanced age can have both immune and autonomic nervous problems that potentially lead to pre-existing pulmonary dysfunction and higher infection risk, increasing the probability of contracting COVID-19. A lifestyle change involving moderate-intensity exercise has the potential to protect against SARS-CoV-2 through strengthening the immune system. In addition to a potential protective measure against SARS-CoV-2, exercise has been shown to improve quality-of-life (QoL) in PD patients. Recent studies provide evidence of exercise as both neuroprotective and neuroplastic. This article is a literature review investigating the role exercise plays in modifying the immune system, improving health outcomes in PwP, and potentially acting as a protective measure against SARS-Cov-2 infection. We conclude that exercise, when correctly performed, improves QoL and outcomes in PwP, and that the enhanced immune response from moderate-intensity exercise could potentially offer additional protection against COVID-19.

## 1. Introduction

### 1.1. Parkinson’s Disease

Parkinson’s disease (PD) is the second most common neurodegenerative disorder in the older adult population [1,2,3,4]. Approximately 60,000 new PD cases are diagnosed each year in the United States, adding to the one million people who presently have PD. Currently, there are a total of ~7–10 million people worldwide living with the disease. The symptoms of PD begin due to the progressive loss of dopamine-producing neurons in the brain’s substantia nigra pars compacta region. Lewy bodies, which are denatured aggregates of the protein alpha-synuclein, are found in these neuronal cells, the formation of which promotes neuronal cell dysfunction and death [1,2,3,4]. PD primarily presents as a movement disorder (symptoms including rigidity, slowness of movement, postural instability, and resting tremor); however, numerous comorbid nonmotor symptoms include depression, psychosis, sleep disorders, dementia, and apathy [5,6].

The majority of PD cases occur sporadically and are usually of unknown cause, except for specific genetic mutations that promote early-onset of the disease. The complex etiology and pathogenesis of PD include neuroinflammation, immune system dysfunction, mitochondrial dysfunction, genetic mutation, oxidative stress, protein aggregation, and multifactorial environmental factors [2,3,7]. Furthermore, this implies that a multipronged therapeutic (or intervention) strategy is needed to halt or slow PD progression [4,8,9]. The traditional approach for treating PD begins with a pharmacologic dopamine replacement strategy. The first line of therapy is either carbidopa/levodopa or a dopamine agonist. Some drugs prolong the lifetime of endogenous dopamine. As the disorder progresses, deep brain stimulation surgery is helpful when medication responses are no longer able to stabilize the symptoms [4,8,9]. Complementary and alternative medicine (CAM) and integrative medicine approaches are also used by many to improve brain and overall health [10]. Different forms of exercise are neuroprotective and neuroplastic besides exercising being used to strengthen the quality-of-life (QoL) in PD [9,10,11,12,13].

### 1.2. COVID-19

The emergence of severe acute respiratory syndrome coronavirus 2 (SARS-CoV-2), or COVID-19, has introduced a new global public health crisis [14,15,16]. SARS-CoV-2 likely originated in Wuhan, Hubei, China, in late 2019. Importantly, COVID-19 was declared a pandemic by the World Health Organization (WHO) in early 2020. Like SARS and Middle East Respiratory Syndrome (MERS), SARS-CoV-2 is a beta coronavirus thought to have a zoonotic origin [14,15,16,17].

SARS-CoV-2 preferentially interacts with type I and type II pneumocytes in the lungs. SARS-CoV-2 binds to the angiotensin-converting enzyme 2 (ACE2) receptor on these cells [16]. To transport viral RNA into the host cell, the mammalian transmembrane protease, serine 2 (TMPRSS2), digests viral proteins following SARS-CoV-2/ACE2 binding to create membrane fusion between virion and host [18]. As expected, due to target cell specificity, the most frequent clinical symptoms of COVID-19 infection include fever, cough, and dyspnea [15,19]. It is mild in the majority, but in more severe cases of COVID-19, patients progress to pneumonia with sepsis, resulting in discernible hypoxemia and likely a requirement for mechanical ventilation [15,18,20]. This exaggerated pro-inflammatory state could lead to acute respiratory distress syndrome with cytokine storm syndrome (CSS) [21,22]. A dysregulated immune response likely causes the occurrence of CSS in individual patients.

The COVID-19 pandemic has altered the world and its various lifestyles [23,24,25,26]. We are currently waiting for the development of vaccines and other antiviral treatments against SARS-CoV-2. In an attempt to reduce SARS-CoV-2 transmission, we locked down entire communities economically and socially, reduced ease of travel across country borders, introduced the concept of social distancing, mandated the wearing of facial masks, and suggested frequent hand washing and sanitizing of home and work areas. Interestingly, there is substantial evidence that moderate exercise boosts the immune system [27,28,29,30,31], which could potentially provide some resistance to SARS-CoV-2.

### 1.3. A Potential Sinister Predicament Includes Parkinson’s Disease in Older Adults and COVID-19

There is evidence for two groups of people who will likely have worse outcomes following COVID-19 infection. These include older adults and those with pre-existing medical conditions (chronic kidney disease, chronic obstructive pulmonary disease, obesity, serious cardiovascular disease, severe immunocompromised state, type 2 diabetes, sickle cell anemia, and frailty), or both [32,33,34,35,36,37]. An additional possibility is that people-with-Parkinson’s (PwP) would be at increased risk of worse outcomes from COVID-19 since PD is more common in older adults, and PwP can have compromised respiratory and immune states [38,39,40].

**Figure 1:**
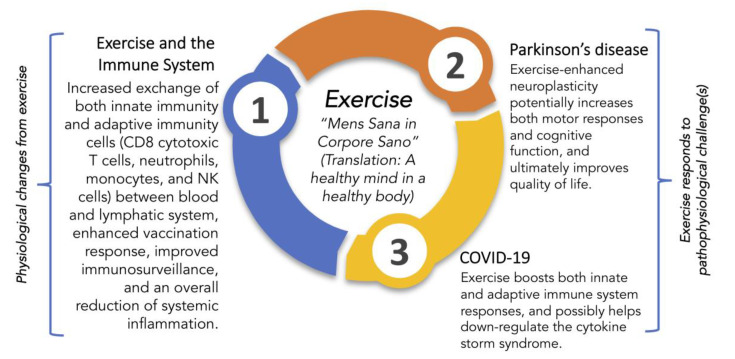
An overview of some health benefits of exercise by modulation of the immune system and putative protective roles for exercise in encounters with Parkinson’s disease and SARS-CoV-2. Juvenal’s centuries-old dictum to seek a healthy mind in a healthy body is from [42].

In just a few months, we have gained a tremendous amount of understanding regarding COVID-19. While significant effort is focused on finding either a cure or effective therapies for COVID-19, to date, treatment is primarily supportive, and the role of antiviral protection remains in testing. The lack of treatment options leads to pressure to find other ways to combat an infection that disproportionately affects older adults, the immunocompromised, and those with pre-existing medical conditions. Recently, we suggested that vitamin D_3_ was a feasible option [41]. Another possible option is exercise. The goal of this review is to outline research elucidating the relationship between exercise and beneficial changes in the immune system and then apply this information to demonstrate how exercise improves QoL and outcomes in PD and possibly helps resist COVID-19 infection (Figure 1).

## 2. Exercise and the Immune System

### 2.1. Defining Exercise

Exercise is a physical activity that requires effort, and it is usually carried out to sustain or improve health and fitness [43,44]. We define low-intensity exercise as activity that increases your heart rate up to 40–50% of your maximum heart rate (MHR). This would include a casual walk with a pet dog or walking up a few flights of steps instead of taking the elevator. We define moderate-intensity (or simply moderate) exercise as physical activity for ≤60 min that increases your heart rate to 50–70% of your MHR. Moderate-intensity exercise should feel somewhat hard; that is, your breathing quickens, but you are not out of breath. You develop a light sweat after about 10 min of activity, but you can carry on a conversation (from the Mayo Clinic [45] and the American Heart Association [46]). We define high-intensity (also called vigorous) exercise as physical activity where your heart rate increases to 70–85% of your MHR. The change found in the immune system from exercise depends on the duration and the intensity of exercise [27,29,30].

### 2.2. Defining the Immune System

**Figure 2:**
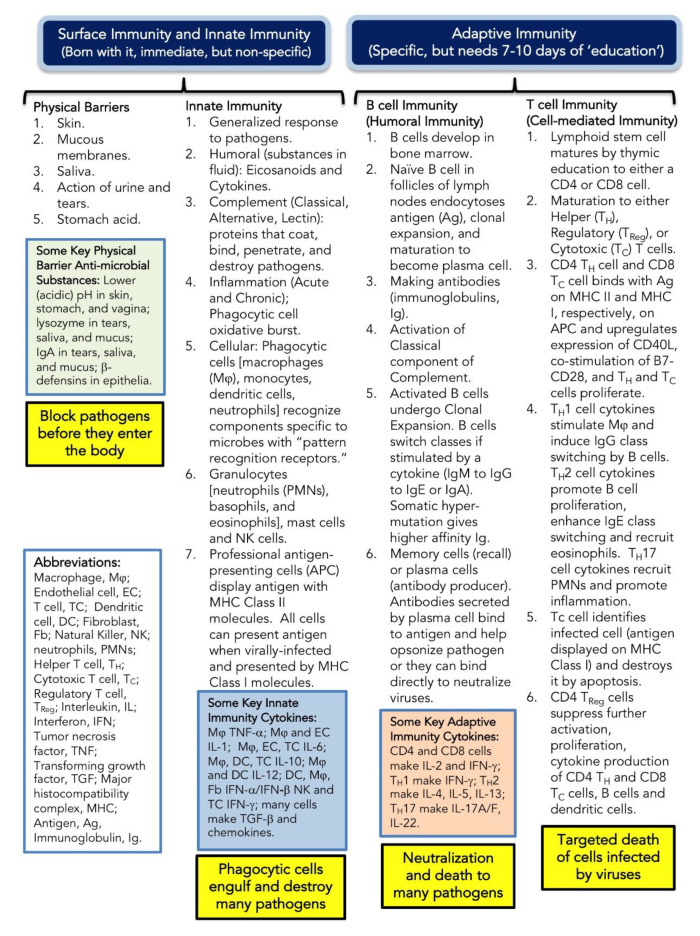
Overview of the immune system.

Defining the immune system is somewhat daunting, and describing it in detail is beyond the scope of this review [47]. The immune system protects the body against infection and non-native substances through intricate pathways that ultimately distinguish everything foreign from nonforeign within a given person. We present an overview of the main components of the immune system, especially those central to this review, outlining the interplay of white blood cells and some of the many biological responses that activate and control the immune system (Figure 2).

An essential aspect of the immune system is inflammation, which is the immune system’s response to infection, injury, and toxic compounds (Figure 2). We consider the inflammatory process to be a host-defense response mechanism. Unfortunately, inflammation, if not controlled, can result in host morbidity and mortality. In severe COVID-19, there is potential for an exponential release of pro-inflammatory cytokines (termed Cytokine Storm Syndrome), which can result in uncontrolled inflammation and result in multi-organ damage with frequent progression to death [21,22].

### 2.3. Moderate Exercise Equals a Balance between the Anti-Inflammatory and Pro-Inflammatory Responses

**Figure 3:**
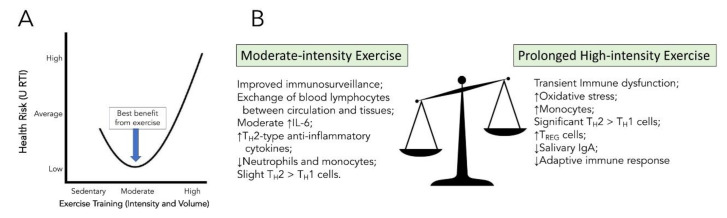
J-shaped curve of upper respiratory tract infections (URTI) risk versus exercise training (Panel **A**) and balancing the positive benefits and negative effects of exercise intensity on the immune system (Panel **B**).

Both cross-sectional and longitudinal data have shown that individuals who participate in moderate-intensity exercise self-report less upper respiratory tract infections (URTI). Additionally, in animal models, those exposed to moderate-intensity exercise before symptom presentation have lower rates of mortality [48,49]. A prospective study in humans assessing the dose–response relationship between moderate walking/running energy expenditure and pneumonia, respiratory disease, and aspiration pneumonia mortality, found that higher doses of moderate exercise decreased mortality in all three categories [50]. In contrast, many studies have shown that prolonged high-intensity aerobic exercise leads to increased death from a viral respiratory infection and over suppression of the immune response [51,52,53]. These findings have led to the development of the ”J-shaped” hypothesis that articulates this balance between exercise dose and URTI risk (Figure 3).

The actions of cytokines are contained within two broad categories, either a pro-inflammatory or anti-inflammatory induction based on highly coordinated immune-stimulatory responses [54]. A common theme across groups, though, is that an increased level of fitness due to exercise training is associated with lower circulating concentrations of pro-inflammatory cytokines and higher circulating concentration of anti-inflammatory cytokines [55]. IL-6 is explicitly associated with higher than baseline levels of skeletal muscle production following prolonged exercise, though exercise duration is a critical factor in determining postexercise plasma IL-6 amplitude [56,57,58,59]. In humans, the infusion of IL-6 increases levels of known anti-inflammatory modulators such as plasma cortisol, IL-1 receptor antagonist, and IL-10. It also attenuates LPS-stimulated production of the pro-inflammatory cytokine TNF-α in both cultured monocytes and in vivo in humans [58,59,60,61].

The cells of the innate immune system express various pattern recognition receptors (PRRs) that recognize specific pathogen-associated molecular patterns (PAMPs) to mount a highly coordinated immune response [62]. Toll-like Receptors (TLRs) are a type of PRR that can be found on the cells of the innate immune system (monocytes/macrophages, neutrophils, dendritic cells, and natural killer cells), playing a significant role in the induction of a pro-inflammatory immune response [62]. Interestingly, Flynn et al., showed that there was a blunted response of LPS-stimulated blood cultures collected from older women following ten weeks of resistive exercise training [63,64]. TLR4 mRNA was lower in the women who participated in the training versus the women who did not [63,64]. Further studies have been conducted in elderly populations, showing a reduction in both TLR2 and TLR4 protein following resistance-training programs. Collectively, these findings provide additional evidence for an exercise-induced anti-inflammatory status.

As mentioned in Figure 1, exercise promotes some wide-ranging changes in both innate and adaptive cells, including increased phagocytic cell action, increased T cell proliferation, and redistribution of lymphocytes to further strengthen the defense system [27]. Lowder et al. investigated the immunologic mechanism as to why moderate-intensity exercise produces the most beneficial health response [53]. In the study, mice that performed 20–30 min of moderate-intensity treadmill exercise had significantly reduced total cellular infiltration and IFN-γ gene expression in lung tissue compared to the sedentary group of animals. The exercise group of mice also had a shift in cytokine profile within the lungs, predominately favoring T_H_2 cells > T_H_1 cells in comparison to sedentary controls. In support of this finding, there was a two-fold increase in IL-4 within exercise mice, a cytokine that promotes differentiation of naïve T_H_ cells to T_H_2 [53]. Although these results show a slight preference for anti-inflammatory T_H_2 cells in the lungs, the T_H_1 cellular response is diminished but still able to perform immunosurveillance. The T_H_2 skewing that initiates the adaptive immune response and anti-inflammatory modulators, though, balances this pro-inflammatory reaction. In comparison, there is a highly polarized T_H_2 to T_H_1 profile shift during high-intensity, prolonged exercise, representing a severe reduction in a pro-inflammatory response and a large increase in an anti-inflammatory one. Martin et al. also found that moderate-intensity exercise shifts both innate and adaptive immune systems in slight favor of T_H_2 cells, providing a more favorable reduction in T_H_1 cell response to viral pathogens in comparison to high-intensity exercise [28].

Furthermore, there is a substantial increase in oxidative stress under prolonged high-intensity exercise conditions [65], and the concentration of salivary IgA, which serves to inactivate foreign substances, is reduced in subjects of prolonged high-intensity exercise duration [66].

In summary, when exposing the body to moderate-intensity exercise training, there is the best balance between the pro- and anti-inflammatory immune benefits: an initial increase in immunosurveillance and an overall reduction in excessive local pro-inflammatory markers (Figure 3).

## 3. Exercise and Parkinson’s Disease Protection

### 3.1. Role of Exercise in Neuroprotection

Aspects of both the innate and adaptive immune systems are active in PD, and they contribute to the pathogenesis of the disorder [67,68]. In the periphery, circulating monocytes, tissue macrophages, and dendrites have an essential role in maintaining the body’s cellular environment through phagocytosis, antigen processing and presentation, and the production of cytokines and chemokines [47]. The CNS monocyte equivalent is the microglia cell, the resident innate immune cells of the brain [67]. There are two subsets of microglia cells: the M1 pro-inflammatory phenotype and the M2 anti-inflammatory phenotype.

**Figure 4:**
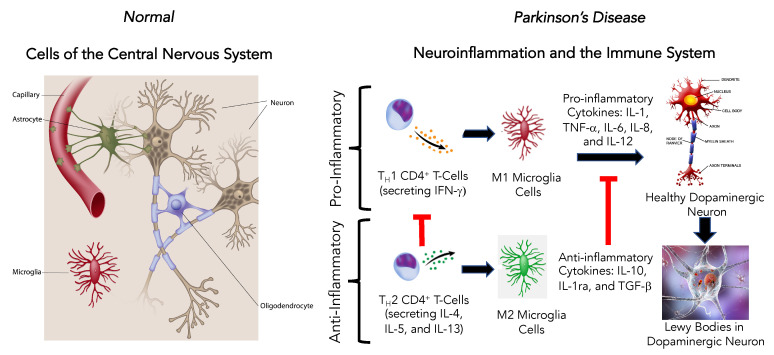
Effect of neuroinflammation and the immune system to promote Parkinson’s disease in the central nervous system.

Emerging research has begun exploring the role of neuroinflammation in the maintenance and progression of PD (Figure 4) [67,68]. There is evidence of sustained pro-inflammatory microglia cell activation and T cell infiltration, and higher levels of pro-inflammatory cytokines (TNF-α, IL-1β, interferon-γ, IL-6, nitric oxide synthase) in both human PD patients and animal models of PD (Figure 4). As PD progresses, α-synuclein (α-Syn), ATP, and metalloproteinase-3 are released from degenerating dopaminergic neurons to enhance microglia cell activation and neuroinflammation, which contribute to dopaminergic neural cell degeneration [69,70].

Two hallmarks of PD are the progressive dopaminergic neuronal cell loss and the aggregation of α-Syn to form Lewy bodies in the surviving affected neurons [71]. This accumulation of α-Syn has been identified in both sporadic and familial PD [67]. As a consequence of this α-Syn aggregation, a protective cerebral innate and adaptive inflammatory response occurs as activated pro-inflammatory microglia cells (M1) accumulate around the α-Syn aggregations and inflammatory pathways mediated by Toll-like receptor-2 (TLR2) and the activation of myeloid differentiation factor-88 (MyD88), nuclear transcription factor-kB (NF-kB), TRAF6, and TAK-1 [72].

Given the role neuroinflammation plays in the initiation, maintenance, and progression of PD, there is a need for the development of neuroprotective therapies that target the inflammatory pathways. Previous studies illustrating the anti-inflammatory effect of moderate to vigorous exercise have led to research into exercise training as a potential therapy.

Palasz et al. investigated the effect of long-term physical activity initiated before and after PD induction in the MPTP-mouse model [73]. The treadmill exercise consisted of the mice running at 10 cm/s for 5 min, 15 cm/s for 5 min, 20 cm/s for 20 min, 25 cm/s for 5 min, and 20 cm/s for 5 min. This protocol is moderate- to high-intensity exercise based on the definition of moderate treadmill exercise in mice [74,75,76]. They reported that in both early and late-onset exercise (treadmill) training, there was (a) preservation of dopaminergic neurons in substantia nigra pars compacta (SNpc) and ventral tegmental area (VTA), (b) an increase in brain-derived neurotrophic factor in the midbrain and glial cell line-derived neurotrophic factor in the striatum, and (c) mitigation of the pro-inflammatory response in the SNpc and VTA [73].

Zhou et al. investigated the neuroprotective effect of a running wheel exercise in PD mouse models [77]. The mice had open running wheel access for three months; it could be calculated that the mice ran 5 miles/day. Their results showed that (i) exercise significantly improves motor and cognitive function; (ii) prevents α-Syn oligomer accumulation in the brain while increasing monomers and dimers in plasma; and (iii) significantly increases levels of DJ-1, Hsp70, and BDNF, which are neuroprotective substances [77].

Two other studies investigating the neuroprotective effects of exercise in mice PD induction models collectively found in swimming [78] and endurance exercise [72] groups: (i) decreased levels of reactive oxygen species, (ii) significant reductions in α-Syn protein along with diminished pro-inflammatory cytokines (TNF-α and IL-β), and (iii) decreased activation of TLR2 and its subsequent downstream signaling cascades (MyD88, TRAF6, and TAK1) [72,78]. The *treadmill* exercise for this study occurred at 10 m/min for 60 min/day [72]. This is moderate intensity based on the definition of moderate treadmill exercise in mice [74,75,76]. The results of Jang et al. [72] imply that exercise is neuroprotective in PD from the reduction in α-Syn that reduces inflammation. Goes et al. [78] suggests that exercise promotes both anti-oxidation and anti-inflammatory properties in chemical-induced models of PD.

These animal models collectively reinforce the evidence that exercise’s anti-inflammatory properties can potentially be harnessed in a neuroprotective role, mitigating the immune system’s neuroinflammation that is characteristic of PD. Importantly, mouse models provide mechanistic insight into how exercise promotes change at the molecular, cellular, and neural network levels [79]. They emphasize the importance of additional studies to better understand the mechanistic changes induced by exercise in humans as well.

In human models, it has been shown that sustained moderate exercise can improve QoL in PD and is likely to help to down-regulate neuroinflammation [12,72,78,80,81,82,83,84,85,86,87,88,89,90,91]. Going from moderate- to high-intensity exercise (in animal models) further reduces neuroinflammation, which implies neuroprotection [73,77]. O’Callaghan et al. showed that high-intensity interval training (HIIT) exercise significantly increased the neuroprotective substance brain-derived neurotrophic factor (BDNF) compared to both moderate-intensity exercise and controls [92]. Some of the issues addressed in PwP studies revolve around improving QoL, motor vs. nonmotor skills, and measured changes in gait, balance, stiffness, axial mobility, and cognitive improvements. The majority of PwP studies suggest that sustained moderate-intensity exercise will ultimately improve QoL in PD and likely reduce neuroinflammation. Hopefully, future studies will continue to differentiate moderate- vs. high-intensity exercise in regard to impact on neuroinflammation and QoL.

### 3.2. Role of Exercise in Neuroplasticity

Neuroplasticity is the capacity of brain cells to adapt, change, and form new connections in response to intrinsic and extrinsic factors. Despite age-related changes in cognitive function and brain structure with aging, functional neuroimaging studies have shown that the brain maintains the ability to increase its breadth of functioning with age [11,93,94].

Regular exercise is associated with the release of endogenous neurotrophins, such as glia-derived neurotrophin (GDNF) that are related to synaptic plasticity, enhanced cognitive ability, learning, and memory [12]. Most evidence of exercise-induced neuroplasticity is derived from studies involving animal models exposed to forced use, task-specific, and high-intensity exercise. Tillerson et al. found that in rats with unilateral dopamine depletion, forced use of the impaired forelimb spared asymmetrical motor impairment and decreased the extent of dopamine neuronal degeneration [95]. Cohen et al. completed a follow-up to this study [96]. They found that in the forced use limb, in addition to spared asymmetrical motor impairment, there is an increase in GDNF in the striatum in response to forced exercise [96].

Several other studies replicated these data using different exercise paradigms such as treadmill exercise, environmental enrichment, and voluntary running. Exceptions to these data replication have suggested that the type of motor training (skilled vs. aerobic) affects the degree of protection PD [12]. Steiner et al. [97] found that PD model rats living in an enriched environment with physical activity for seven weeks showed a significant increase in the number of NG2-positive and GFAP-positive cells in the substantia nigra of the basal ganglia, suggesting significant gliosis in response to exercise.

In response to significant neuroprotective evidence in animal models, Farley et al. introduced the principle of intensive amplitude-specific exercise-based therapeutic approaches for humans [12,80,98]. This innovative exercise program named LSVT/BIG was focused on improving disease outcomes in PwP. The loss of dopamine in PD triggers faulty processing, output, and feedback within the basal ganglia (BG). Their hypothesis suggested that instead of bypassing the BG in treatment, targeted amplitude exercise (LSVT/BIG) could be used to harness neuroplasticity to enhance activation of damaged BG pathways and potentially slow their degradation [12,80,98]. An LSVT/BIG-certified Neurologist prescribes LSVT/BIG to the PwP, who then has specialized training sessions with an LSVT/BIG-certified Physical Therapist (PT). After completion of the 4 weeks (4 days/week) of program training, the PwP continues the LSVT/BIG exercises on their own to further address their motor-related problems.

The Berlin LSVT/BIG comparative study randomly assigned mild to moderate PD patients to either supervised LSVT/BIG training (BIG), supervised Nordic Walking (WALK), or nonsupervised exercises (HOME) [99]. Following 16 weeks of therapy and exercise, there was a 5.05 improvement in Unified Parkinson Disease Rating Scale (UPDRS) score in the BIG group, whereas there was a 0.58 and 1.68 increase, respectively, in UPDRS scores in the WALK and HOME groups [99]. A retrospective study by Isaacson et al. of PwP taking part in the LSVT/BIG program found significant improvements in dual-tasked tests (specifically measuring mobility and cognitive performance while performing two tasks simultaneously) [100]. These studies with LSVT/BIG suggest that intensive amplitude-directed exercise programs, when directed by a knowledgeable PT, are capable of achieving activity-dependent neuroplasticity and motor learning in PwP.

Schenkman et al. found that changes in motor symptoms, measured with UPDRS scores, were significantly smaller for PwP using a high-intensity treadmill exercise routine than moderate-intensity exercise and control groups [101]. A significant trend is the incorporation and utilization of exercise in optimizing the care of PwP [9,102], and there are numerous PD-directed exercise programs/routines [10,87,88,90,103,104,105,106]. Expectantly, future studies will continue to compare the impact of moderate-intensity and high-intensity exercise programs on neuroplasticity in combination with the investigation into the overall health impact of exercise in PD.

Understanding how exercise promotes neuroplasticity in humans has been difficult. A few obstacles include which biological markers are the most useful for measuring neuroplasticity and what imaging techniques/tools would be most helpful to document changes in the neural networks [107]. A clinical trial [108] and a feasibility study [109] for exercise-induced neuroplasticity in PD are currently underway.

## 4. Exercise and COVID-19 Protection

The research connection between moderate exercise and reduced occurrence of URTI suggests moderate exercise as a potential protective measure against infection with COVID-19 and as a possible mitigator of symptoms if COVID-19 is acquired [48,49,50,51,52,53]. We suggest that the following four responses of the immune system to moderate exercise could act as potential nonpharmacological prophylaxis for COVID-19.

### 4.1. Short-Term Pro-Inflammatory Induction Postexercise and Long-Term Increases in Anti-Inflammatory Modulators Prevent Acute/Chronic Inflammatory Tissue Damage

We described earlier in the review that intense exercise can result in immunosuppression. In contrast, moderate-intensity exercise can improve immune function, potentially reducing the risk and severity of viral respiratory infections [27,53,65,66]. When exposing the body to moderate exercise training, there is an initial increase in immunosurveillance and an overall reduction in excessive local inflammation and inflammatory markers. While moderate exercise shifts the cytokine profile slightly to T_H_2 cells, an active T_H_1 cell response is still present. A T_H_1 inflammatory immune response leads to the up-regulation of pro-inflammatory cytokines, especially IFN-γ. A T_H_1 modulated cytotoxic response is needed for early antiviral clearance activity, and a T_H_2 response is necessary for the production of viral neutralizing antibodies, infection eradication, and modulation to prevent over inflammation. In contrast to moderate exercise, vigorous exercise creates an even more polarized T_H_2 to T_H_1 cytokine profile, oversuppressing the immune system. These findings provide evidence for positive viral respiratory outcomes in individuals who participate in moderate versus vigorous exercise, and the balance of moderate exercise can maintain between the positive benefits of pro- and anti-inflammation when fighting a viral infection such as COVID-19.

### 4.2. Restoration of Damaged Lung Tissue

Moderate aerobic exercise has the potential to restore the elasticity and strength of lung tissue and respiratory muscles. Park and Han found that in women over the age of 65, aerobic exercise on a treadmill increased Forced Vital Capacity (FVC) and Forced Expiratory Volume in 1 s (FEV_1_), indicating an improvement in markers of alveolar compliance and elasticity and reduced resistance to airflow in the respiratory tract [110]. Taskin et al. also found that moderate aerobic exercise increased the strength of respiratory muscles, inspiratory muscle performance, and diminished the perception of dyspnea in patients with ankylosing spondylitis [111]. These findings provide evidence for potential protective and therapeutic effects of moderate exercise on lung integrity when faced with the risk of COVID-19.

### 4.3. Prevention and Reduction in Reactive Oxygen Species

Moderate aerobic exercise works to reduce oxidative stress, a frequent instigator of acute and chronic lung injury, through the decreased production of reactive oxygen species [112,113]. Toledo et al. found evidence for the attenuation of pulmonary disease development as a result of moderate exercise. They found that moderate aerobic exercise diminished the decrease in pulmonary elastin induced by cigarette smoke exposure and significantly decreased the presence of reactive oxygen species in bronchiolar lavage fluid, along with other indicators of inflammatory mediated damage [114].

A leading cause of death in patients with COVID-19 is Acute Respiratory Distress Syndrome (ARDS). The acute lung injury in ARDS results from high levels of neutrophil activation and accumulation of reactive oxygen species and pro-inflammatory mediators. Extracellular Superoxide Dismutase (EcSOD) prevents oxidative stress and damage through extracellular scavenging of superoxide anions [115]. Yan and Spalding described the role of exercise in increasing EcSOD, initially enhancing EcSOD expression in skeletal muscle and then redistributing to lung tissue. This role is a possible preventative or therapeutic option for reducing risk and severity of ARDS in COVID-19 [115].

### 4.4. Targeted Decrease in the Main Health Risk Factors of COVID-19

Increasing an individual’s capacity for aerobic exercise can act as a potential mitigator for the main risk factors of COVID-19: aging, hypertension, diabetes, and heart problems. Each of these conditions can be lessened or improved through exercise [112,116,117,118,119,120].

Another critical risk factor in determining health factors for COVID-19 is frailty; a marker defined using the 7-point Clinical Frailty Scale [37]. In 2019, Hewitt et al. found that worsening frailty, at any age, was predictive of worse health outcomes in a population sampled for acute surgical admissions [121]. Frailty has recently become a prognostic marker informing acute COVID-19 care pathway decisions amid ventilator and intensive care unit bed shortages [37]. Hewitt et al. set out to explore the efficacy of using frailty in this context, utilizing mortality and length of stay data for patients with COVID-19 at ten hospitals in the UK and one in Italy [37]. They found that frailty was linked both to earlier deaths and more extended stays in the hospital from COVID-19 and that these outcomes were worse if frailty increased, regardless of age or other comorbidities [37]. Frailty can also be present in PwP, further complicating the clinical decisions for these patients [122]. Importantly, exercise has been found to reduce frailty when examined in older adults who had impairments in physical performance and oxygen uptake [123,124,125,126]. These studies indicate that frailty treated with individualized exercise programs that include aerobic exercise (low to potentially moderate intensity, depending on the health status of the patient) and strength and flexibility training is most promising. These results imply that through exercise, frailty, a risk factor for COVID-19, could potentially be reduced with improved physiological capacity (i.e., increased mobility and oxygen uptake) and some enhanced immunological functions.

## 5. Conclusions

Exercise and physical activity improve the health of the aging processes in older adults [127,128,129,130]. The Active People, Healthy Nation™ recommendation is the same for adults (ages 18–64) and older adults (65 years and older) for substantial health benefits. The recommendation includes 150 to 300 min per week of moderate-intensity exercise, or 75 min to 150 min a week of high-intensity aerobic activity, or some combination of moderate- and high-intensity aerobic activity [44]. The recommendation also includes at least two days/week of muscle-strengthening action [44]. Activities that improve balance for older adults, such as standing on one foot, is also encouraged [44].

Regular physical exercise has numerous beneficial health effects in PD, including the impact on the immune system and the potential to reduce neuroinflammation (which would be neuroprotective) and promote neuroplasticity. Persistent moderate-intensity exercise by PwP is a treatment strategy to improve the QoL and likely reduce neuroinflammation. Using more demanding moderate- to high-intensity exercise programs (examples include PWR!Moves, Rock Steady Boxing, Dance for PD, stationary bike, and power-walking with walking poles) [10,87,88,90,91,92,101,103,104,105,106,109], with sustained use, could eventually promote neuroplasticity in PwP.

Finally, research has shown that the immune response is strengthened from moderate-intensity exercise. Thus, exercise may provide a nonpharmacological strategy if COVID-19 infects one or if one wants to protect themselves from infection [31]. Older adults, with or without PD, are more susceptible to SARS-CoV-2 viral infection, and moderate exercise may help to improve the immune response to COVID-19 infection. Moderate-intensity exercise may also help boost the immune system response to the COVID-19 vaccination when it becomes available.
